# The risk factors of the occurrence of inguinal hernia in ESRD patients receiving dialysis treatment: An observational study using national health insurance research database

**DOI:** 10.1097/MD.0000000000031794

**Published:** 2022-12-09

**Authors:** Pin-Han Chiu, Jui-Ming Liu, Ming-Li Hsieh, Wei-Tang Kao, Kai-Jie Yu, See-Tong Pang, Po-Hung Lin

**Affiliations:** a Department of Surgery, Chang Gung Memorial Hospital at Linkou, Taoyuan, Taiwan; b Department of Urology, Taoyuan General Hospital, Ministry of Health and Welfare, Taoyuan, Taiwan; c Division of Urology, Department of Surgery, Chang Gung Memorial Hospital at Linkou, Taoyuan, Taiwan; d Research Center of Urology and Kidney, Taipei Medical University (TMU-RCUK), Taipei, Taiwan; e Department of Urology, Shuang Ho Hospital, Taipei Medical University, New Taipei City, Taiwan; f Graduate Institute of Clinical Medical Science, College of Medicine, Chang Gung University, Taoyuan, Taiwan; g School of Medicine, College of Medicine, Chang Gung University, Taoyuan, Taiwan.

**Keywords:** ESRD, hemodialysis, inguinal hernia occurrence, peritoneal dialysis, risk factors

## Abstract

With the quickly growing population of patients receiving dialysis treatment in Taiwan in recent years, concerns about whether more incidence of inguinal hernia exists in dialysis patients are increasing. In Taiwan, peritoneal dialysis (PD) and hemodialysis (HD) are the 2 most common dialysis types. Therefore, the relationship between dialysis type and inguinal hernia occurrence needs to be evaluated and compared. Our retrospective cohort study included a study population total of 3891 patients diagnosed with end stage renal disease (ESRD) under the HD or PD procedure from 2001 to 2009 from the Longitudinal Health Insurance Database. Also, International Statistical Classification of Diseases and Related Health Problems 9th Revision codes were used to identify ESRD and hernia occurrence. Cox proportional-hazards regression model was applied to measure the risk factors to the hernia occurrence. During the follow-up periods of 3 years, the number of hernia occurrences was 44 (1.13%), 1 (0.03%), and 8 (0.21%) with inguinal, femoral, and ventral hernias, respectively. Only the dialysis type revealed significantly increased hernia risk because PD would increase hernia risk 7 times (adjusted hazard ratio [aHR] = 6.98, 95% CI = 3.59–13.25) than HD. If the patients received PD and shifted to HD later, the risk of hernia was 5 times (aHR = 4.98, 95% CI = 2.29–10.85) than patients with HD. Patients with ESRD receiving PD or PD-HD shift were risk factors of inguinal hernia occurrence. The results may help clinicians increase the alert of possible risk factors and complications at the beginning of dialysis treatment in patients with ESRD.

## 1. Introduction

Inguinal hernia, which accounts for 75% of abdominal wall hernia, has a mortality risk of 2.7% and 3% in men and women, respectively.^[1,2]^ Nearly 500,000 cases seek medical attention annually. Moreover, the frequency of surgical repair of inguinal hernia varies among countries with rates ranging from 10 per 100,000 to 28 per 100,000 of the population in the UK and USA, respectively.^[2–4]^

Increasing age, the male gender, smoking, physical exertion, chronic obstructive pulmonary disease (COPD) and peritoneal dialysis (PD) have been reported as risk factors of inguinal hernia.^[1,5–11]^ The cause of inguinal hernia is multifactorial and is, however, mainly due to intraabdominal pressure increase.^[12]^

With the quickly growing population of patients with dialysis in Taiwan in recent years, there are increasing concerns about whether more incidence of inguinal hernia exists in patients with dialysis treatment. In our study, we evaluated the relationship of inguinal hernia occurrence between the 2 dialysis types, PD and hemodialysis (HD), of which the end-stage renal disease (ESRD) patients receiving the most in Taiwan. Furthermore, patients’ age, gender and underlying diseases were also analyzed in our current study.

## 2. Methods

### 2.1. Database

The Longitudinal Health Insurance Database (LHID) was used to conduct this retrospective cohort study. The LHID 2000 is derived from the National Institutes of Health, which sampled 1 million representatives from all recipients of the National Health Insurance Research Database (NHIRD) in Taiwan in 2000.

The NHIRD had documented all the medical information of patients, including diagnosis, prescription, medical procedure, and hospital records from 2001 to 2009. The LHID is part of the NHIRD, and specific patients could be followed according to the long-term tracking of specific diseases and more than 99% sampling coverage of the population in Taiwan. The study has been approved by the Institutional Review Board of Chang-Gung Memorial Hospital (103-2071B). All the data in the database was delinked to the individual, and no personal data could be identified; therefore, informed consent was not necessary.

### 2.2. Study population

Patients diagnosed with ESRD were included under the HD or PD procedure from 2001 to 2009. Also, diagnoses of ESRD based on the International Statistical Classification of Diseases and Related Health Problems 9th Revision (ICD-9) code of 585 were combined with the claim codes of HD (58001C, 58002CB, 58018C-58025C, 58027C, and 58029C) or with the claim codes of PD (58002C, 58009A, 58009B, 58010A, 58010B, 58011A, 58011B, 58011AB, 58011C, 58012A, 58012B, 58017A, 58017B, 58017C, 58026C, and 58028C). All patients with ESRD received catastrophic illness certificates, which are subject to government review and enable medical fee reductions.

### 2.3. Clinical outcomes

The hernia occurrence was the clinical outcome of the current study and included 3 types of hernia (e.g., inguinal, femoral, and ventral hernias). Hernia diagnoses were also identified based on the ICD-9 coding as inguinal (550.00-550.03, 550.10-550.13, and 550.90-550.93), femoral (551.00-551.03, 552.00-552.03, and 553.00-553.03), and ventral (551.20, 551.21, 551.29, 552.20, 552.21, 552.29, 553.20, 553.21, and 553.29) hernias by the ICD-9 codes. To ensure the effect of dialysis type on hernia occurrence, only newly developed hernia 3 months after dialysis initiation was considered as related to dialysis, and up to 3 years were followed at most after the diagnosis of ESRD. Moreover, the criteria wherein participants who had diagnoses of HD or PD > 1 month before the study period had been employed to exclude patients with emergent dialysis.

### 2.4. Statistical analysis

The Cox proportional-hazards regression model was used to measure the risk of different dialysis types to the hernia occurrence and calculated the adjusted hazard ratios and 95% confidence intervals (CIs). Moreover, *P* < .05 was considered statistically significant. All data were analyzed by the SAS statistical software package (version 9.4, SAS Institute, Cary, NC) to express the mean standard deviation and investigate factors associated with inguinal hernia development in patients with dialysis.

## 3. Results

**Figure 1.**
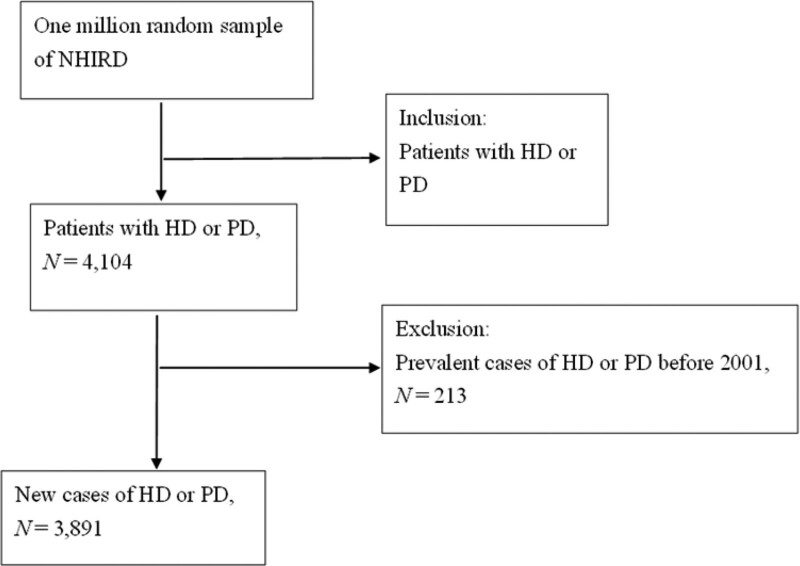
Flowchart of recruitment of subjects from 1 million random samples of NHIRD from 2001 to 2009 in Taiwan. HD = hemodialysis, PD = peritoneal dialysis, NHIRD = the National Health Insurance Research Database.

The patient enrollment protocol is shown in Fig. 1. Firstly, ESRD was defined as patients with ICD-9 code 585 and had received HD or PD for > 1 month. Of the patients, 4104 were retrieved from a population database of 1 million. Secondly, 213 patients diagnosed with ESRD before 2001 were excluded because newly diagnosed patients with ESRD were intended to be included. Finally, 3891 patients who fulfilled the ESRD criteria were enrolled for analysis.

The demographic data of 3891 patients are presented in Table 1. The mean age of patients with ESRD was 62.19, and around 60% of patients were older than 60 years old. No gender difference was noted. Among the 3891 patients, 3417 initially received HD where 265 patients received PD and 209 patients initially received PD and shift to HD later.

**Table 1:** Demographic data of the whole population.

Variable	*N* (%)
No. of cases	3891
Mean age (standard deviation)	62.19 (14.49)
Age	
≤30	124 (3.19)
31–40	170 (4.37)
41–50	508 (13.06)
51–60	766 (19.69)
61–70	886 (22.77)
71–80	787 (20.23)
≥81	650 (16.71)
Gender	
Male	1919 (49.32)
Female	1972 (50.68)
Underlying disease#	
Hypertension	3363 (86.43)
Diabetes mellitus	2321 (59.65)
Chronic liver disease	1080 (27.76)
Coronary heart disease	1712 (44.00)
Cerebral vascular accident	1134 (29.14)
Heart failure	1096 (28.17)
COPD	907 (23.31)
Constipation	1211 (31.12)
Obesity	30 (0.77)
BPH	611 (15.70)
HD PD shift	
HD only	3417 (87.82)
PD only	265 (6.81)
Shift	209 (5.37)
Hernia	
Inguinal hernia	44 (1.13)
Femoral hernia	1 (0.03)
Ventral hernia	8 (0.21)
No	3838 (98.64)

BPH = benign prostate hyperplasia, COPD = chronic obstructive pulmonary disease, HD = hemodialysis, PD = peritoneal dialysis.

# Only for underlying disease diagnosed more than one year before the occurrence of hernia.

During the follow-up period of 3 years after ESRD diagnosis, the number of hernia occurrences was 44 (1.13%), 1 (0.03%), and 8 (0.21%) with inguinal, femoral, and ventral hernias, respectively.

Patients with ESRD were divided into with or without hernia groups to analyze possible factors related to hernia occurrence. Demographic data, comorbidities, and dialysis type were analyzed (Table 2).

**Table 2:** Relationship between hernia development and demographic data of patients.

Variable	Nonhernia	Hernia	*P* value	
Mean age (standard deviation)	62.24 (14.45)	58.90 (16.69)	.0967	
Gender				
Male	1885 (49.11)	34 (64.15)	.0297	*
Female	1953 (50.89)	19 (35.85)		
Underlying disease#				
Hypertension	3317 (86.43)	46 (86.79)	.9382	
Diabetes mellitus	2296 (59.82)	25 (47.17)	.0622	
Chronic liver disease	1067 (27.80)	13 (24.53)	.5972	
Coronary heart disease	1690 (44.03)	22 (41.51)	.7131	
Cerebral vascular accident	1123 (29.26)	11 (20.75)	.1760	
Heart failure	1084 (28.24)	12 (22.64)	.3678	
COPD	892 (23.24)	15 (28.30)	.3868	
Constipation	1198 (31.21)	13 (24.53)	.2964	
BPH	598 (15.58)	13 (24.53)	.0754	
HD PD shift				
HD only	3388 (88.28)	29 (54.72)	<.0001	***
PD only	250 (6.51)	15 (28.30)		
Shift	200 (5.21)	9 (16.98)		

BPH = benign prostate hyperplasia, COPD = chronic obstructive pulmonary disease, HD = hemodialysis, PD = peritoneal dialysis.

# Only for underlying disease diagnosed more than one year before the occurrence of hernia.

* *p* < .05

*** *p* < .0001.

Mean age between the 2 groups and underlying diseases, such as hypertension, diabetes mellitus, chronic liver disease, coronary heart disease, cerebral vascular accident, heart failure, COPD, constipation, and benign prostatic hyperplasia showed no significant difference. Statistically significant differences were found in both male gender and PD type between the 2 groups.

The adjusted hazard ratios of each variable are shown in Table 3. Age, gender, and underlying diseases, such as hypertension, diabetes mellitus, chronic liver disease, coronary heart disease, cerebral vascular accident, heart failure, COPD, constipation, and benign prostatic hyperplasia showed no significant difference. Only the dialysis type revealed significantly increased hernia risk because PD would increase hernia risk 7 times (adjusted hazard ratio = 6.98, 95% CI = 3.59–13.25) than HD. If the patients received PD and shifted to HD later, the risk of hernia was 5 times (adjusted hazard ratio = 4.98, 95% CI = 2.29–10.85) than patients with HD.

**Table 3:** Adjusted hazard ratios of hernia development with 95% confidence intervals of the demographic data.

Variable	Hazard Ratio			Adjusted		
Age	0.99	(0.97, 1.00)		1.00	(0.98, 1.02)	
Gender (Ref: Female)						
Male	1.85	(1.05, 3.24)	*	1.63	(0.87, 3.05)	
Underlying disease						
Hypertension (Ref: No)	1.03	(0.47, 2.29)		1.11	(0.48, 2.57)	
Diabetes Mellitus (Ref: No)	0.60	(0.35, 1.03)		0.78	(0.43, 1.41)	
Chronic liver disease (Ref: No)	0.84	(0.45, 1.58)		0.89	(0.47, 1.69)	
Coronary heart disease (Ref: No)	0.90	(0.52, 1.56)		1.17	(0.63, 2.20)	
Cerebral vascular accident (Ref: No)	0.64	(0.33, 1.23)		0.71	(0.35, 1.44)	
Heart failure (Ref: No)	0.75	(0.39, 1.42)		0.91	(0.46, 1.83)	
COPD (Ref: No)	1.30	(0.72, 2.37)		1.51	(0.80, 2.86)	
Constipation (Ref: No)	0.72	(0.38, 1.34)		0.81	(0.41, 1.56)	
BPH (Ref: No)	1.75	(0.94, 3.28)		1.73	(0.81, 3.69)	
Type (Ref: HD)						
PD	6.90	(3.70, 12.88)	***	6.89	(3.59, 13.25)	***
Shift	5.15	(2.44, 10.88)	***	4.98	(2.29, 10.85)	***

BPH = benign prostate hyperplasia, COPD = chronic obstructive pulmonary disease, HD = hemodialysis, PD = peritoneal dialysis.

* *p* < .05

*** *p* < .0001.

## 4. Discussion

The Taiwan NHIRD was used in the present study to investigate the factors associated with the hernia incidence in patients with ESRD for the high-prevalence dialysis population in Taiwanese society. Of all the factors investigated, male patients and patients receiving PD were found to may have a higher risk of hernia, and patients receiving PD had 7 times hernia risks than patients receiving HD.

The risk factors for developing a primary hernia include increased age, male gender, chronic cough, polycystic kidney disease, and PD.^[1,3,5,6,11,13]^ Also, a major risk factor of groin hernia development is a family history of groin hernia, which is associated with 8 times the risk.^[1]^ Moreover, higher body mass index (BMI) patients were more likely to suffer from inguinal hernia than low BMI patients.^[7]^ Whether heavy lifting is also a risk factor remains controversial. The current study showed that male gender and PD were the risk factors for inguinal hernia development in patients with ESRD. Furthermore, the current findings are comparable with previous reports^[8,14]^ although others observed no complications.^[12,15]^

Abdominal wall hernia, especially inguinal hernia, which accounts for 83.08% of all hernia in the present study, is a common mechanical PD complication.^[16]^ Patients receiving PD tend to have more pressure in the abdomen due to the presence of dialysis fluids in the abdomen cavity.^[17]^ The increased intraabdominal pressure is proportional to the infused peritoneal volume and is also related to the higher incidence of hernia development.^[11,16,18–20]^ The current study showed that patients receiving PD had 7 times greater risk of developing hernia than HD.

Male gender was also a risk factor for hernia occurrence.^[7–9]^ According to several previous studies, a higher incidence of inguinal hernia was observed to be 10 times more common in men than in women.^[1,3,7,10]^ The inguinal canal, where the spermatic cord enters the scrotum, is supposed to act as a pathway from the abdomen wall to the genitalia.^[1]^ For men, this canal is weaker versus women due to testicles descending after birth and the canal not closing up properly. It leaves a gap for tissue to push through the abdomen wall. As investigated in the current study, males are more susceptible to develop hernias than females because 34 (64.15%) of males versus 19 (35.85%) of females had an inguinal hernia, which was statistically significant (*P* = .0297).

Obesity, though not statistically significant in the current study, is often linked to the rising frequency of inguinal hernia due to elevating abdominal pressure. Several previous articles illustrated that BMI is associated with the incidence of inguinal hernia occurrence.^[3,7,8,21]^ Some that indicated higher BMI is a protective factor for inguinal hernia compared to patients with lower BMI, and the temporarily hypothetical explanation was the strengthened abdominal adipose tissue or musculature proved to have a stronger barrier against herniation, while others thought patients with higher BMI are more likely to suffer from inguinal hernia than low BMI.^[3,7,8,14,22,23]^ However, limited data were collected in the current study, and more studies are needed to determine whether the association between inguinal hernia and obesity is true and the effect of body fat and abdominal muscle distribution needs to be evaluated further as well.

The current study has some limitations. First, details of some patients’ demographic data cannot be retrieved from the database (e.g. BMI, infusion volume of dialysis solution, hernia occurrence, and patient’s compliance to dialysis). Therefore, the association of hernia risk and these factors cannot be further investigated. Second, the NHIRD is based on the medical fee claim data. Thus, the details of examinations or results of studies cannot be found. Nevertheless, multiple criteria (e.g., ICD code in addition to the coding of dialysis to increase the accuracy of data coding) were used. Third, we did not investigate the different types of PD, as the automated peritoneal dialysis (APD) and continuous ambulatory peritoneal dialysis (CAPD) since there is no associated data in NHIRD. The intra-abdomen pressure (IAP) is different in patients receiving APD or CAPD and may affect the occurrence of hernia. However, either for APD or CAPD the IAP is higher than patients receiving HD, and the results still support the hypothesis that higher IAP would increase the risk of hernia formation. Fourth, only the risk factors of hernia occurrence in patients with dialysis and without further study in severity or treatments or hernia recurrence were investigated. Further study may be necessary to evaluate if patients with PD have a related severe or high recurrence rate of hernia. The strengths of our present study include a large sample size and inclusiveness. Since almost all patients in Taiwan are enrolled in NHIRD of Taiwan, we could greatly enhance the external validity of the findings. Also, we found that increasing risk of inguinal hernia occurrence in ESRD patients as long as receiving PD before and we explained possible reasons behind its significant influence of PD on the risk forming inguinal hernia. Our findings may alert clinical doctors to do more health education and personal care of PD patients to prevent potential inguinal hernia occurrence.

## 5. Conclusion

In summary, the findings from the current study help figure out the relationship between the incidence of inguinal hernia and different types of dialysis. Patients with ESRD receiving PD had 7 times greater risk of hernia development compared with patients receiving HD and whether patients receiving PD or PD-HD shift were risk factors of inguinal hernia in patients with ESRD. The current study may help clinicians increase the alert of possible risk factors and complications at the beginning of dialysis treatment and provide more health education to prevent further abdominal complications for ESRD patients with dialysis treatment in Taiwan.

## Author contributions

**Conceptualization:** Pin-Han Chiu, Jui-Ming Liu, Ming-Li Hsieh, See-Tong Pang, Po-Hung Lin.

**Data curation:** Pin-Han Chiu, Wei-Tang Kao, Kai-Jie Yu, Po-Hung Lin.

**Formal analysis:** Jui-Ming Liu, Wei-Tang Kao, Po-Hung Lin.

**Methodology:** Po-Hung Lin.

**Project administration:** Ming-Li Hsieh, Kai-Jie Yu.

**Resources:** Kai-Jie Yu, See-Tong Pang.

**Supervision:** Jui-Ming Liu, Ming-Li Hsieh, See-Tong Pang, Po-Hung Lin.

**Validation:** Kai-Jie Yu.

**Writing—original draft:** Pin-Han Chiu, Jui-Ming Liu.

**Writing—review and editing:** Po-Hung Lin.
